# Socio-environmental and endocrine influences on developmental and caste-regulatory gene expression in the eusocial termite *Reticulitermes flavipes*

**DOI:** 10.1186/1471-2199-11-28

**Published:** 2010-04-23

**Authors:** Matthew R Tarver, Xuguo Zhou, Michael E Scharf

**Affiliations:** 1 Department of Entomology and Nematology, University of Florida, Gainesville FL, USA; 2 Formosan Subterranean Termite Research Unit, USDA-ARS-SRRC, New Orleans LA, USA; 3 Department of Entomology, University of Kentucky, Lexington, KY, USA

## Abstract

**Background:**

Strict regulation of caste differentiation, at the molecular level, is thought to be important to maintain social structure in insect societies. Previously, a number of extrinsic and intrinsic factors have been shown to influence caste composition in termite colonies. One important factor is the influence of nestmates; in particular, soldier termites are known to inhibit hormone-dependent worker-to-soldier differentiation. However, soldier influences on nestmates at the molecular level are virtually unknown. Here, to test the hypothesis that soldiers can influence nestmate gene expression, we investigated the impact of four treatments on whole-body gene expression in totipotent *Reticulitermes flavipes *workers: (i) juvenile hormone III (JHIII; a morphogenetic hormone), (ii) soldier head extracts (SHE), (iii) JHIII+SHE, and (iv) live soldiers.

**Results:**

Using quantitative-real-time PCR we determined the expression patterns of 49 previously identified candidate genes in response to the four treatments at assay days 1, 5, and 10. Thirty-eight total genes from three categories (chemical production/degradation, hemolymph protein, and developmental) showed significant differential expression among treatments. Most importantly, SHE and live soldier treatments had a significant impact on a number of genes from families known to play roles in insect development, supporting previous findings and hypotheses that soldiers regulate nestmate caste differentiation via terpene primer pheromones contained in their heads.

**Conclusions:**

This research provides new insights into the impacts that socio-environmental factors (JH, soldiers, primer pheromones) can have on termite gene expression and caste differentiation, and reveals a number of socially-relevant genes for investigation in subsequent caste differentiation research.

## Background

Phenotypic plasticity can be described as the production of variable phenotypes from a single genotype based on conditions encountered throughout an organism's development [1]. Phenotypic plasticity can be divided into gradual or discrete polyphenisms. Reaction norms are phenotypically graded responses to environmental factors. Polyphenisms, occur when two or more discrete alternative phenotypes occur without intermediate forms [2].

Social insects have evolved to produce and use multiple alternate phenotypes (i.e., polyphenism) to accomplish a wide range of tasks within their colonies. Castes are phenotypically and behaviorally discrete individuals that cooperate to perform colony tasks [3]. Termites are hemimetabolous social insects that utilize castes to meet various needs within the colony. Most termite colonies are made up of three distinct castes: *workers*, *soldiers*, and *reproductives *[4]. All termite eggs, except when a rare genetic component might be involved [5], are considered totipotent, and most evidence supports that castes differentiate based on gene expression responses to intrinsic and extrinsic factors. The research presented here examined gene expression responses in worker termites to both intrinsic and extrinsic factors.

All castes except soldiers and reproductives retain the ability to molt, while soldiers and reproductives are considered terminally developed [6]. Caste differentiation can proceed along two routes; imaginal (winged) or apterous (wingless). The first developmental branch is the point at which larvae differentiate into apterous workers or imaginal nymphs. Nymphs can either (i) regress into worker-like "pseudergates", (ii) differentiate into fully winged and eyed adult alates that disperse and found new colonies, or (iii) differentiate into winged and eyed non-dispersive brachypterous reproductives that serve as supplemental reproductives within the colony. Workers are totipotent in that they can (i) undergo status quo worker-to-worker molts, (ii) differentiate into soldiers (after passing through an intermediate presoldier stage), or (iii) differentiate into apterous and eyeless neotenic reproductives that serve supplementary reproductive roles [6-8].

The entire complement of intrinsic and extrinsic factors that dictate each of the developmental switches in termites, and how they interact, are yet to be fully understood. Examples of intrinsic factors include juvenile hormone (JH), storage proteins, and nutrition; whereas examples of extrinsic factors include primer pheromones, temperature, food quality, nestmates (soldiers and reproductives), and season [9-19].

Phenotypic divergence from the worker to the soldier caste can be mediated by multiple JH-related factors. For example, elevated JH titers in workers are correlated with presoldier differentiation [20-22]. Additionally, the presence of soldiers has been shown to inhibit the formation of new soldiers, implying that soldier termites produce inhibitory factors that cause reduced responsiveness to JH or reduced JH biosynthesis in nestmates [15,16,23,24]. This inhibition is presumed to be caused by soldier-derived primer pheromones [10,25,26]. Primer pheromones are defined as chemical messengers that are passed among individuals and trigger physiological responses in recipients [27]. Recently, *R. flavipes *soldier head extracts (SHE), when applied in combination with juvenile hormone III (JH III), were found to enhance presoldier production compared to JH III alone [19]. Two major components of *R. flavipes *SHE are γ-cadinene and its aldehyde γ-cadinenal; they represent the first candidate primer pheromones to be identified from termites [19]. Interestingly, SHE alone does not impact presoldier formation [19]. Also, while the SHE blend is active at influencing JH-dependent presoldier differentiation, the individual impacts of its constituents and whether they are being actively released or absorbed has yet to be determined.

Functional genomics is a powerful approach for elucidating the functions of genes, including genes that mediate pheromone and hormone action [28]. Transcript levels generally correlate with the physiological demand for the product they produce; thus, changes in transcript abundance can reveal genes that are most important in relation to a stimulus [28]. Such an approach has been used to elucidate the chemical ecology of the bark beetle (*Ips pini*) [28-31] and the honeybee (*Apis mellifera*) [32]. Similarly, the use of functional genomics in studies of termite caste regulation can help to better understand potential primer pheromone function as well as the influences of intrinsic and extrinsic factors on caste differentiation.

The central goal of this research was to use a functional genomics approach to identify candidate caste-regulatory genes from *R. flavipes *workers that potentially mediate hormonal and soldier primer pheromone signaling. Four treatments were tested on isolated groups of worker termites: (i) JH III alone, (ii) soldier head extract (SHE) alone, (iii) JH III + SHE, and (iv) live soldiers. These treatments represent key intrinsic and extrinsic/socio-environmental factors that are thought to impact soldier development in totipotent workers. Our central hypothesis was that these four treatments will be associated with the differential expression of key genes through time, and that key responsive genes will play significant roles in meditating caste differentiation and/or caste-regulatory signaling. Our approach involved determining the impacts of the four treatments on both phenotypic caste differentiation and the expression of forty-nine candidate and reference genes during the first 10 days of differentiation from worker to presoldier, with subsequent validation of reference genes and post-hoc analyses to identify genes with significant differential expression among treatments. Here, we identify and discuss a number of responsive genes from three categories (chemical production/degradation, hemolymph protein, and developmental) with significant links to caste differentiation.

## Results

### Phenotypic responses

**Figure 1:**
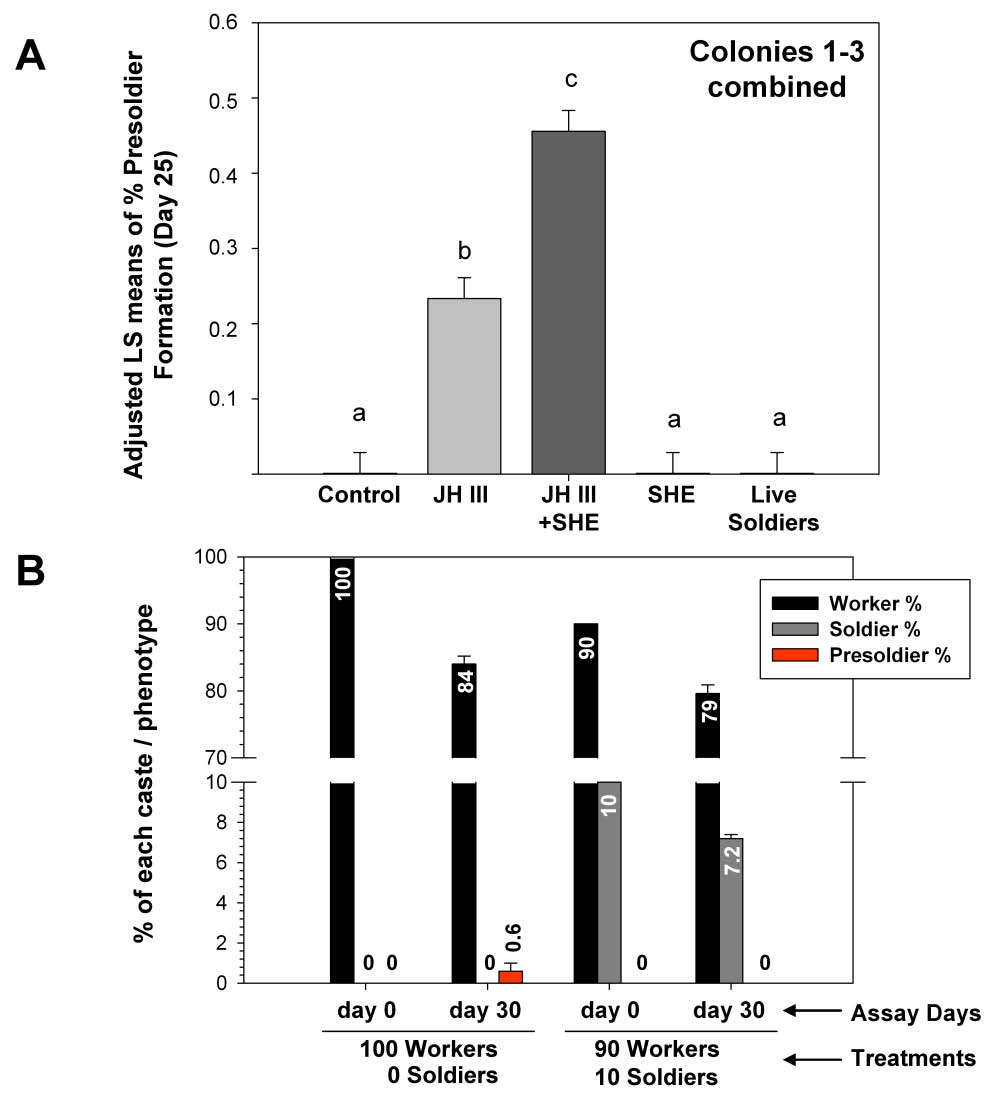
**Impact of semiochemical and socio-environmental treatments on soldier caste differentiation**. (A) Cumulative presoldier formation through Day 25 of assays that compared five different treatments: untreated controls, JH III, JH III+SHE, SHE, and live soldiers for three different colonies. Each replicate dish (n = 5 per colony) contained 15 workers. Results for the three colonies were pooled for analysis. Adjusted LS means are shown; bars with the same letter are not significantly different (*P *< 0.05). (B) Post-hoc presoldier induction assays conducted using 100 termites per replicate dish (n = 5). The two treatments that were examined included starting compositions of 100 workers + 0 soldiers, or 90 workers + 10 soldiers. No presoldiers formed after 30 d. in treatments that included soldiers at the beginning of assays. This finding reveals that *R. flavipes *soldiers indeed are capable of inhibiting worker differentiation to presoldiers.

Phenotypic bioassays (Fig. 1A) showed that the combination of JH III + SHE significantly increased presoldier development when compared to JH III alone. A two-way ANOVA and adjusted LS means were used for analysis (whole model F = 24.092, df = 14, P < 0.0001; treatment F = 54.32, df = 4, P < 0.0001; colony F = 24.140, df = 2, P < 0.0001; treatment*colony F = 11.513, df = 8, P < 0.0001). Variation was observed between the different colonies tested, with Colony 1 showing the greatest presoldier induction response to JH III (40%) and JH III+SHE (80%). But, as seen in previous research [19], the overall trend was the same in that JH III+SHE increased presoldier differentiation compared to JH III alone and no presoldiers formed in the acetone-treated controls, SHE-alone treatments, or live soldier treatments (Fig. 1A).

Because no phenotypic effects were observed with live soldier treatments in the small-format dish assays noted above, we conducted *post-hoc *presoldier induction assays using larger groups of workers (Fig. 1B). Our objective was to determine if soldiers could inhibit natural presoldier formation in the absence of ectopic JH, using greater numbers of workers (100 termites) over a longer period of time (30 days). Two treatments were tested: (1) 100 workers + 0 soldiers, and (2) 90 workers + 10 soldiers. Interestingly, no presoldiers formed after 30 d. in treatments that included 10% soldiers at the beginning of assays; and conversely, presoldiers appeared only in the treatments that included 100% workers at the beginning of assays. This finding verifies that *R. flavipes *soldiers are capable of inhibiting worker-to-presoldier differentiation, and provides evidence that is directly supportive of the soldier and SHE impacts on gene expression presented below.

### Reference gene selection

To accurately determine relative gene expression in totipotent workers, we chose three reference genes that had stable expression across all treatments and colonies (*Stero-1*, *LIM*, and *Mev-1*). These reference genes were selected by comparing the standard deviation of the raw Ct values for all 49 genes across treatments (Additional file 1: Table S1). This determination is important because it allows normalization of the expression of target genes (n = 46) to reference genes (n = 3) that have stable expression across all treatments and colonies.

### Gene expression overview

All target and reference genes investigated in this study have been annotated based on significant translated identity to insect sequences deposited in the Genbank nr and EST databases. Full-length gene names are provided in Additional file 2: Table S2. All reported gene expression data represent the average of three independently sampled and replicated *R. flavipes *colonies. Gene expression changes in response to all treatments were determined via qRT-PCR. To identify genes with significant differential expression across treatments, two-way ANOVAs were used with adjusted LS means and FDR correction on normalized CT (ΔCT) values (Additional files 3, 4, 5, 6: Tables S3, S4, S5, S6). Additionally, gene expression at three days (1, 5 and 10) was analyzed separately using the ANOVA procedure noted above. For a large proportion of the genes tested there was a significant colony effect. This was to be expected because (i) there was also a significant colony effect in the phenotypic bioassay and (ii) the colonies tested each have different mitochondrial haplotypes (see later). Colony effects were compensated for by using adjusted LS means in the analysis.

**Figure 2:**
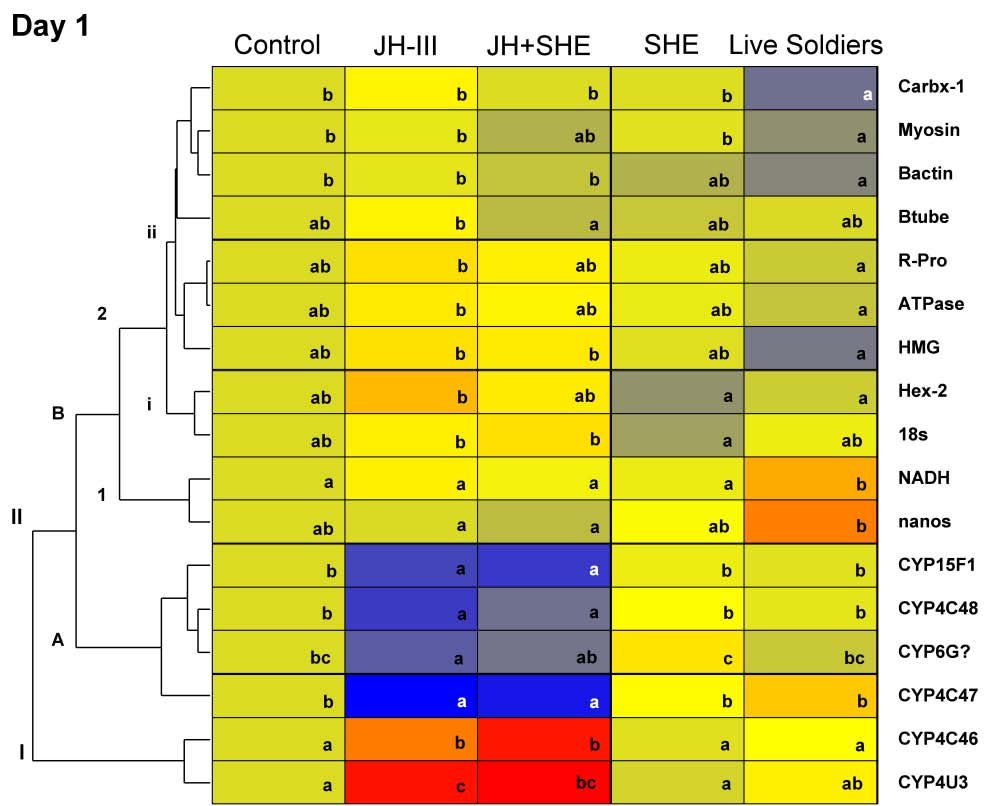
**Expression changes for significant genes in termite workers in response to hormonal, semiochemical and socio-environmental treatments after 1 day**. Results shown represent the relative expression values of significant differentially expressed genes under five different treatments: control, JH III, JH III+SHE, SHE, and live soldiers after one day; Blue boxes represent genes that are down-regulated while red boxes represent genes that are up-regulated. Boxes with the same letter within a row are not significantly different (FDR). Dendrograms at the left group genes by similar expression pattern.

**Figure 3:**
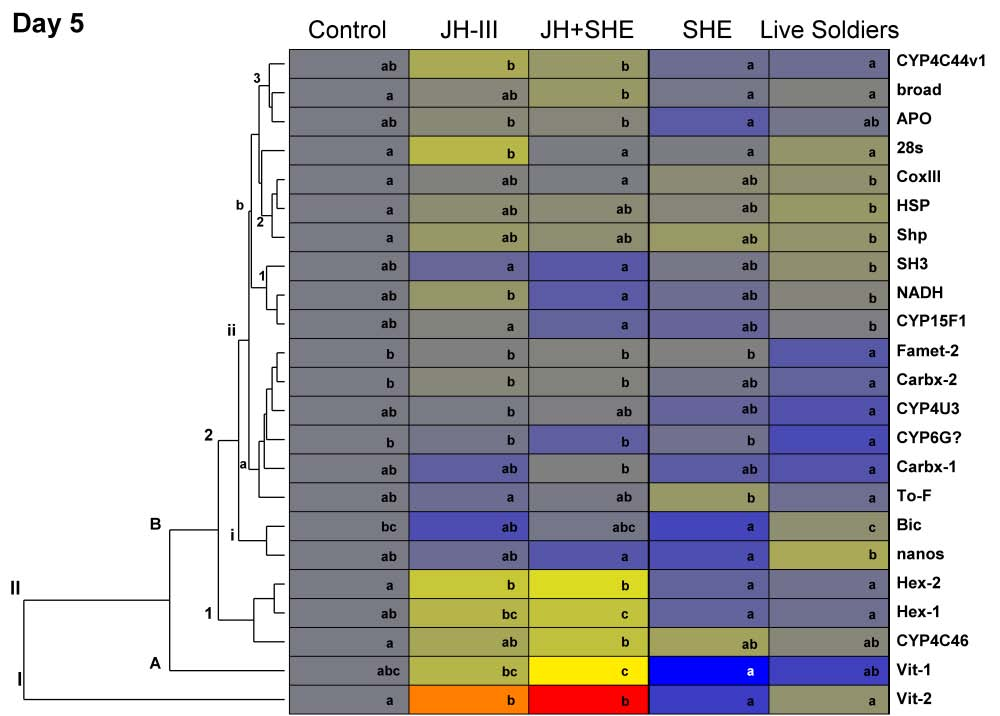
**Expression changes for significant genes in termite workers in response to hormonal, semiochemical and socio-environmental treatments after 5 days**. See Fig. 2 caption for details.

**Figure 4:**
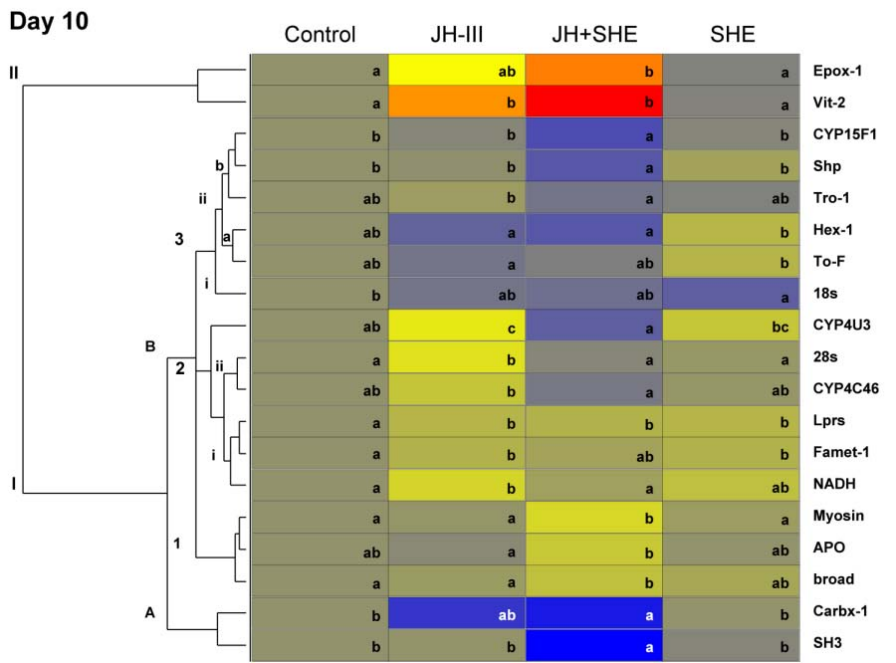
**Expression changes for significant genes in termite workers in response to hormonal, semiochemical and socio-environmental treatments after 10 days**. See Fig. 2 caption for details.

To easily visualize gene expression responses, genes showing significant expression changes across treatments were organized by day into heat maps (Fig. 2, 3, 4). Genes with similar expression profiles are horizontally clustered together. By clustering genes in this manner we are able to identify groups of genes that respond similarly and putatively belong to the same gene networks (also see Additional file 7: Table S7).

### Gene expression: day 1

As shown in the Day 1 heat map (Fig. 2), 17 out of the 46 genes that were tested showed significant differences in their expression across treatments (Additional file 4: Table S4). Day 1 receives focus here because we presume Day 1 responsive genes to be important immediate-early responders. Three main clusters of genes were identified, with sub groupings of genes in some clusters. Genes in group **IIB **overall were affected by SHE and live solder treatments, with **IIB2ii **genes *Carbx-1*, *Myosin*, *B-actin*, *β-tube*, *R-Pro*, *ATPase*, and *HMG *all being down-regulated with live soldiers. Genes in group **IIB1**, *NADH *and *nanos*, were up-regulated in live soldier treatments. Group **IIB2i **genes *Hex-2 *and *18s *were down-regulated in SHE treatments. The P450 protein coding genes in group **IIA**, *CYP15F1*, *CYP4C48*, *CYP6G?*, and *CYP4C47 *were down regulated with JH III and JH III+SHE treatments, while group **I **genes, *CYP4C46 *and *CYP4U3*, were up-regulated with JH III. These Day 1 results reveal a number of early response genes in totipotent workers that are both up and down-regulated in response to the different treatments. Perhaps most importantly, a number of P450 genes that may play roles in semiochemical or hormone processing were differentially expressed among treatments at this early time point.

### Gene expression: day 5

Five days into assays, 23 genes showed significant differential expression among the five treatments (Fig. 3, Additional file 5: Table S5). A larger number of genes showed significant variation in expression at this point compared with Days 1 and 10, with the majority of the genes showing down-regulated responses to most treatments. Genes in group **IIB2iib3**, *CYP4C44v1*, *broad*, and *APO *had a slight expression increase with JH, while being down-regulated with SHE and live soldier treatments. Group **IIB2iib2 **genes, *CoxIII*, *HSP*, and *Shp *displayed an up-regulation with live soldier treatments. Genes *SH3*, *NADH *and *CYP15F1*, in group **IIB2iib1**, were down-regulated with JH+SHE and SHE treatments. Group **IIB2iia **genes, *Famet-2*, *Carbx-1*, *CYP4U3*, *Carbx-2*, and *To-F *were all down-regulated with live soldier treatments. *Bic *and *nanos*, in group **IIB2i**, were down-regulated with JH III, JH III+SHE and SHE treatments. Genes that clustered into group **IIB1**, *Hex-2*, *Hex-1*, and *CYP4C46 *were up-regulated with JH III and JH+SHE treatments. Finally, two hemolymph protein coding genes, *Vit-1 *(**IIA**) and *Vit-2 *(**I**) were up-regulated with JH III and JH+SHE and down-regulated with SHE and live soldier treatments. Five days into assays represents the middle of the worker-to-solder differentiation process [8]. Therefore, genes identified at this time point could be playing mid-level signaling roles in the caste differentiation cascade. The hemolymph protein coding genes *Vit-1*, *Vit-2*, *Hex-1 *and *Hex-2*, have been linked to caste differentiation in past research in termites and honey bees [17,18,33-38]. Thus, their differential expression during the worker-to-presoldier differentiation process was expected, and serves to validate our approach for determining gene expression during differentiation.

### Gene expression: day 10

On the last day investigated (Day 10) nineteen genes showed significant variation in expression across treatments (Fig. 4, Additional file 6: Table S6). Live soldier effects were not investigated at this time point due to limitations imposed by the 96-well PCR plate format and an inability to include all treatments for individual genes on a single plate. The group **II **genes *Epox-1 *and *Vit-2 *were up-regulated with JH III and JH+SHE treatments. Genes in group **IB3iib**, *CYP15F1*, *Shp*, and *Tro-1 *were down-regulated with JH+SHE treatment, while *Hex-1 *and *To-F *(**IB3ii**a) were down regulated with JH III and JH+SHE treatments. The putative ribosomal RNA coding *18s *gene was down-regulated in live soldier treatments (**IB3i**). Group IB2ii genes *CYP4U3*, *28s*, and *CYP4C46 *were up-regulated with JH III but down-regulated with JH+SHE treatment, while genes in group **IB2i**, *Lprs*, *Famet-1*, and *NADH *were down-regulated with JH III. Genes that clustered in group **IB1**, *Myosin*, *APO*, and *broad *were up-regulated with JH+SHE treatment. Finally group **IA **genes, *Carbx-1 *and *SH3*, were down-regulated with JH+SHE treatment. These Day 10 results reveal a number of potential late responding genes that are both up- and down-regulated in response to the different treatments. Thus, these late responding genes likely are part of multiple pathways that are involved in the later stages of the worker-to-presoldier differentiation process.

### Uniformly responsive genes and hierarchical clustering

Across days 1, 5 and 10, four genes showed consistent, significant differential expression: *CYP15F1*, *CYP4C46*, *CYP4U3*, and *NADH*. This finding suggests that these four genes are of broad general importance in worker-to-soldier caste differentiation and/or caste regulation/homeostasis.

**Figure 5:**
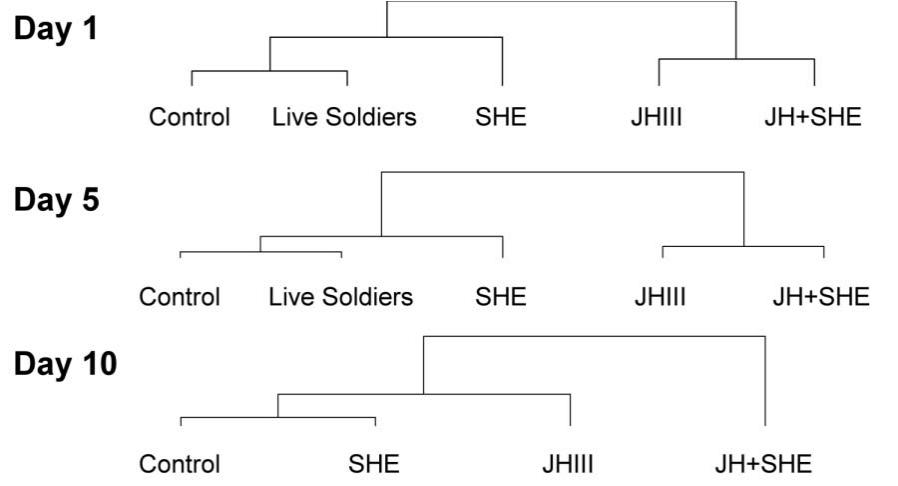
**Hierarchical clustering results of gene expression pattern clustered by treatment**. All three days (A-Day 1, B-Day 5, and C-Day 10) were analyzed separately using the relative expression of each gene by the Euclidean distance metric, with centroid squared linkage.

Finally, gene expression results were hierarchically (vertically) clustered by treatment across days based on the expression patterns of all genes (Fig. 5a,b,c). Results for Day 1 and 5 are similar with control and live soldier treatments clustering together, and JH III and JH+SHE treatments clustering together. Day 10 results show a different clustering pattern in which control and SHE treatments cluster together, and the JH and JH+SHE treatments show a more distant relationship. These results suggest that effects of the different treatments on genes and gene networks are not temporally fixed, but change through time.

## Discussion

Social organisms, including hemimetabolous lower termites like *R. flavipes*, utilize phenotypic plasticity to achieve caste polyphenism and division of labor. Because all termite colony members share essentially the same genetic background, they rely on differential gene expression for caste differentiation [3]. The development of termites along alternate caste pathways is regulated by a number of interacting intrinsic and extrinsic factors (e.g., [18]); however, detailed global gene expression responses through the differentiation process have been lacking, and no prior studies have investigated nestmate or primer-pheromone-responsive gene expression in termites.

This study correlates clear phenotypic effects of *R. flavipes *hormones, semiochemicals, and social treatments with patterns of gene expression and reveals potentially important candidate caste-regulatory genes. Changes in expression of several genes having homology to other well-characterized developmental and hormone/semiochemical biotransformation genes were detected in association with the different treatments. Several gene networks apparently important in caste differentiation and social interactions were also identified.

The model bioassay system used here induces changes in phenotype, and gene/protein expression, and has been used repeatedly to monitor and elucidate mechanisms of caste differentiation, specifically the worker-to-soldier transition [17-19,34,39,40]. Here, we investigated the effects of specific hormone/semiochemical (JH III, JH III+SHE, SHE) and socio-environmental conditions (live soldiers) on soldier caste differentiation and gene expression by totipotent termite workers. Although there are certainly other semiochemical and socio-environmental conditions that could play a role in worker-to-soldier differentiation, we focused on the components listed above because they build concisely on preceding work.

Phenotypic assay results were similar to past findings in that JH III induced presoldier formation, JH III+SHE synergistically increased presoldier formation, and SHE alone had no effect on presoldier development [19]. The addition of live soldiers to the bioassay did not impact soldier formation. Because presoldier differentiation only occurs naturally in larger groups of workers over longer periods of time (see Fig. 1B and [21]), our small-scale model bioassay cannot allow for determination of any inhibitory effects by soldiers at the whole organism level. Nonetheless, our results provide good support to the hypothesis that SHE, or a component of it, acts with JH as a primer pheromone to help regulate caste proportions within termite colonies.

This research also monitored phenotypic effects in concert with the expression patterns of multiple genes. This was accomplished with destructive sampling of some assay replicates for RNA isolation, while allowing others to proceed without disturbance. The typical worker-to-presoldier differentiation process takes approximately 15 days. To capture potential expression changes up to apolysis, gene expression levels were monitored at 1, 5, and 10 days post treatment, which are considered early, middle and late time points, respectively, in the presoldier developmental transition. A total of forty-nine genes were investigated across three replicate colonies. Statistically significant genes that passed the FDR cutoff were clustered together based on expression pattern (Fig. 2, 3, 4). As discussed below, three main groups of responsive genes were identified: (i) chemical production/degradation, (i) hemolymph protein coding, and (iii) developmental.

### Chemical production/degradation genes

Chemical production and degradation genes code for enzymes that are potentially responsible for the production and/or degradation of many types of semiochemicals in termites, including hormones such as JH and ecdysone, as well as the soldier head terpenes γ-cadinene and γ-cadinenal. The three groups of genes included in this category are cytochrome P450, hydrolytic, and mevalonate pathway protein-coding genes.

Cytochrome P450s are known for their role in the oxidation of endogenous and xenobiotic substrates including hormones, pheromones, insecticides, and secondary plant compounds [41,42]. Specifically, P450s have been shown to play a role in the biosynthesis and metabolism of morphogenic hormones (JH, ecdysone) and terpenoids [41]. On Day 1, two groups of P450s were differentially expressed. In the first group (IIA), *CYP15F1*, *CYP4C48*, *CYP6G? *and *CYP4C47 *were down-regulated with JH III and JH III+SHE treatments, while in the second group (I), *CYP4C46 *and *CYP4U3 *were up-regulated with JH III and JH III+SHE treatments. This opposite expression profile of the two P450 groups suggests they have different functions, likely acting on multiple substrates.

Past research has identified P450s that play significant roles in JH biosynthesis and degradation in insects. In the cockroach, *Diploptera punctata*, *CYP15A1 *epoxidizes methyl farnesoate to form JH III [43]. In the present study, those P450s that were down-regulated with JH treatment (*CYP15F1*, *CYP4C48*, *CYP6G? *and *CYP4C47*) could have a similar function. Insect P450s have also been shown to play a role in the degradation of JH III, as is the case with *CYP4C7*, which converts JH III to 12-trans-hydroxy JH III in *Diploptera punctata *[44,45]. The group **I **P450s (*CYP4C46 *and *CYP4U3*) that were up-regulated in the present study could be playing this role and/or the group of genes that were down-regulated could be inactivated, potentially blocking the worker-to-soldier transition.

Juvenile hormone metabolism is also potentially mediated by hydrolytic enzymes, including JH esterases and epoxide hydrolases [46]. Three genes having homology to JH esterases and epoxide hydrolases displayed significant expression differences among treatments. *Carbx-1 *has highest homology to a JH esterase of the wood-feeding beetle *Psacothea hilaris *(BAE94685) [47]. The *Carbx-2 *gene has highest homology to a JH esterase of the sawfly *Athalia rosae *(BAD91555). Both the *Carbx-1 *and *Carbx-2 *genes also have significant homology to honey bee JH esterases [48] as described by Mackert et al. [49]. Both genes are expressed in the gut, and thus could be acting on JH acquired via trophallaxis, but also could play digestive roles by hydrolyzing lignin or hemicellulose carboxyl esters [48].

Epoxide hydrolases are known to degrade JH by hydrolyzing the epoxide bond that is formed by CYP15 action as described above. The epoxide hydrolase studied here, *Epox-1*, has significant homology to an *Aedes aegypti *epoxide hydrolase (XP_001651935), among others. If *Epox-1 *is acting as a JH epoxide hydrolase, its observed up regulation could contribute to the degradation or inactivation of any endogenous remaining JH prior to apolysis or ecdysis, which is expected to occur at around day 10 in our model presoldier induction assays [18].

The production of JH and other sesquiterpenes derived from the mevalonate pathway is important to termite colony success, not only for development and caste differentiation, but also for production of defensive chemicals and pheromones that possess a sesquiterpene backbone [29,50]. Both up- and down-regulation of genes in the mevalonate pathway can significantly impact the production of JH and pheromones [30,51]. In the present study, five mevalonate pathway genes were investigated: *Famet-1*, *Famet-2*, *Famet-3*, *Mev-1*, and *HMG*. Two genes homologous to farnesoic acid methyl transferases (*Famet-1*, *Famet-2*) showed differential expression. Farnesoic acid methyl transferase methylates farnesoic acid, producing the immediate JH precursor methyl farnesoate [50]. RNAi-mediated knockdown of this gene in *Tribolium castaneum *has led to reduced JH levels and precocious molting [52]. *R. flavipes Famet-1 *shares strongest homology to a *FAMet *protein from the hymenopteran *Melipona scutellaris *(AM493719) [53]. Our results revealed that JH causes increased *Famet-1 *expression. Increased expression of this gene could theoretically increase JH biosynthesis rates and enable soldier formation. Our results also revealed that the presence of live soldiers down-regulates *Famet-2 *gene expression, which theoretically could lead to reduced JH production and decreased worker-to-soldier differentiation.

In general, these results suggest that JH III causes up-regulation of mevalonate pathway genes, while live soldiers are suppressive. Consistent with our phenotypic bioassay results, suppression of the mevalonate pathway by live soldiers would likely result in reduced pathway products, such as JH, resulting in reduced JH titers and subsequent reductions in soldier caste differentiation.

### Hemolymph protein coding genes

Four hemolymph protein coding genes, *Hex-1*, *Hex-2*, *Vit-1*, and *Vit-2 *showed significant differential expression through all assay days. These four genes are important in caste differentiation and sociobiology for a number of social insects; therefore, it was not surprising that they showed responsiveness in our experiments. The termite hexamerin genes have been shown to act as part of an environmentally responsive socio-regulatory mechanism that affects the activity of JH, possibly limiting its availability [18,33,34,54].

Two other hemolymph protein genes, *Vit-1 *and *Vit-2*, were up-regulated with JH and JH +SHE treatments at Day 5, but only *Vit-2 *was differentially expressed at Day 10. Throughout the experiment, both *Vit-1 *and *Vit-2 *genes displayed a high amount of variability among treatments and replicates. One explanation for such variance is the inclusion of both sexes of worker termites in assays. In most insects, vitellogenin (*Vg*) serves as a female-specific yolk precursor protein that functions in oocyte provisioning. However, *Vg *has also been shown to play a role in social insect caste regulation; for example, *Vg *in female honeybee workers, has been shown to interact with JH. Specifically, higher JH levels and lower *Vg *levels increased the transition from nursing to foraging behavior by worker bees [35], while a reduction of JH delayed the onset of foraging [55]. Honeybee workers with RNAi-suppressed *Vg *levels performed foraging behaviors earlier than untreated workers [36,37]. Nutrition has also been shown to affect *Vg *and JH by regulating the transition from nursing to foraging [56]. Finally, *Vg *has been shown to affect queen honeybee longevity by interacting with insulin signaling [57]. Together, these findings suggest that honeybee *Vg *has been co-opted away from reproduction to serve as a regulator of caste behavioral polyethism [38]. Results of the current study, showing that *Vit-1 *and *Vit-2 *are up-regulated with JH and JH +SHE treatments, but down-regulated with SHE and live soldier treatments, suggest interesting possibilities with respect termite vitellogenin and caste polyphenism.

### Developmental genes

The dramatic morphological change that occurs as worker termites become soldiers requires significant body plan rearrangement [3]. The soldier termite's large mandibles and their associated muscles represent a large change from the smaller head and reduced muscle mass present in worker termites [58,59]. Thus, it is likely that multiple genes are required to coordinate and achieve this transition [60]. Six developmental genes from two groups, cytoskeletal/structural and body-plan, showed significant differential expression in the current study.

The cytoskeletal/structural protein coding gene "*β-tube" *was significantly differentially expressed at Day 1 among treatments. *β-tubulins *are also hormone-responsive and have been linked to the production of ecdysteroids in *Manduca sexta *[61,62]. *α- *and *β-tubulin *genes were also identified in *Bombyx mori *from several EST libraries linked to imaginal wing disk metamorphosis and 20-hydroxyecdysone [63,64], suggesting roles in restructuring during adult wing formation. Our findings suggest potential roles for *R. flavipes β-tube *in either soldier head muscle function or possibly ecdysone-linked developmental-regulatory processes.

A number of developmental/body plan genes also showed significant differential expression. One body plan gene, *broad *(BTB/POZ) [8], which is homologous to broad (*br*) transcription factor genes of the hemimetabolous and holometabolous insects *(Oncopeltus fasciatus *and *T. castaneum*), was up-regulated at Day 5 with JH and JH+SHE treatment and at Day 10 with JH+SHE treatment. Erezylimaz et al. [65] used RNAi to silence the *br *gene in *O. fasciatus*, causing an additional immature molt. Erezylimaz et al. suggested that *br *is required for morphogenesis, and that its expression is regulated by JH. RNAi silencing of *br *in *T. castaneum *caused similar results [66]. If *br *is acting in the same manner in termites, up-regulation of the gene by JH+SHE would promote the worker-to-soldier transition, which is in agreement with phenotypic bioassay results showing increased presoldier formation in the JH+SHE treatment.

## Conclusions

**Figure 6:**
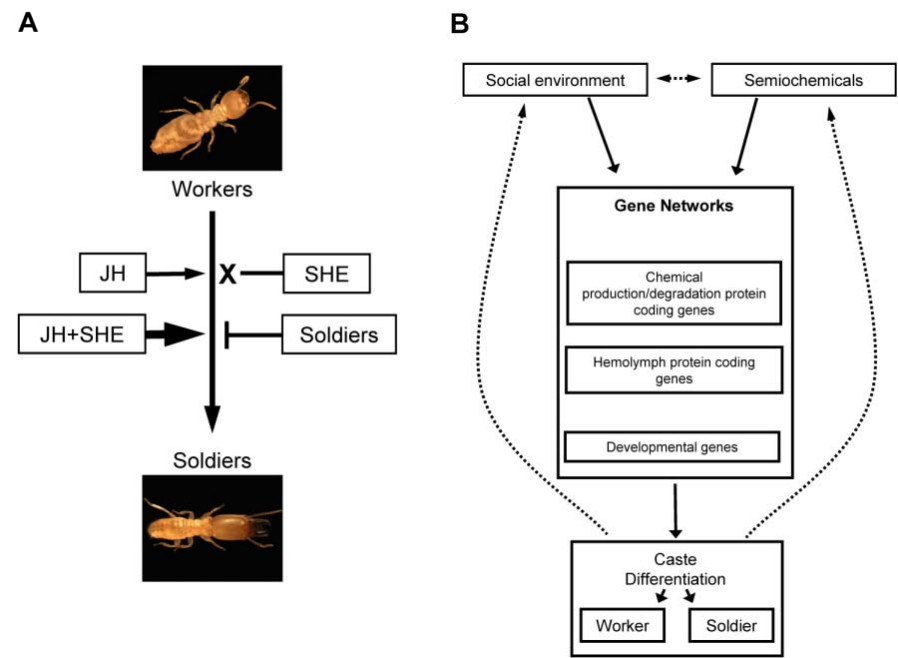
**Diagrams summarizing the influence of socio-environmental and semiochemical factors on caste differentiation**. A) Semiochemical and socio-environmental factors tested and their effects on worker-to-soldier differentiation. JH III and JH+SHE caused an increase in soldier formation, while SHE had no effect on presoldier/soldier formation. Past research [[15,16], personal observations] indicates that soldiers inhibit worker differentiation. B) Diagram representing how socio-environmental and semiochemicals factors might modulate the expression patterns of multiple genes and caste differentiation. Networks including the following gene categories showed significant changes among treatments: chemical production/degradation, hemolymph protein coding, and developmental genes. Dotted lines represent the possible feedback loop when colony worker termites molt into soldiers, the increase in the soldier number consequently inhibits the formation of additional soldiers.

The research presented here demonstrates for the first time the influence that the SHE blend, live soldier caste members, and JH together have on phenotype and gene expression of totipotent termite workers (Fig. 6). To summarize phenotypic assay results (Fig. 6A): (i) JH III induced significant presoldier differentiation, (ii) JH + SHE induced significantly higher levels of presoldier differentiation, (iii) the crude SHE blend by itself did not have any observable phenotypic effects, and (iv) live soldiers inhibited presoldier formation in the absence of ectopic JH. In support of primer pheromone hypotheses initially proposed by Lüscher [67] and further developed by Henderson [68], our results provide the first evidence that the soldier caste has direct impacts on caste-regulatory gene networks, and subsequently, worker caste differentiation. Significant responsive gene categories identified here include chemical production/degradation genes, hemolymph protein coding genes, and developmental genes (Fig. 6b). Past reports (e.g., [16]) and the present research (Fig. 1B) have demonstrated that live soldiers do indeed inhibit natural presoldier formation. These results, in addition to the current gene expression findings, support earlier hypotheses that live soldiers act as part of a negative feedback loop, inhibiting new soldier formation by regulating the expression of genes important for caste differentiation (Fig. 6b) [16,24,68]. Recent findings have further revealed that γ-cadinene and γ-cadinenal levels increase in workers that are held with soldiers (MR Tarver, *unpublished results*), which lends significant strength to the results presented here that show live soldier and SHE impacts on gene expression. The next steps in this research will follow up on these observations by investigating the impacts of pure γ-cadinene and γ-cadinenal on phenotypic caste differentiation and on the expression of responsive genes identified in the current study.

This research provides important new evidence of impacts on nestmate gene expression by live termite soldiers and crude soldier head extracts. While further research is needed to resolve the roles of soldiers and SHE blend components in termite caste regulation (via RNA interference, gene expression localization, further analysis of SHE constituents, investigating impacts of SHE constituents on gene expression, or using whole-genome micro-arrays or next-generation transcriptome sequencing) the findings of this study provide a solid foundation on which to conduct further translational studies.

## Methods

### Termites

*R. flavipes *colonies were collected from different locations near Gainesville, Florida USA. Termites were held in the laboratory for at least two months before use in bioassays. Colonies were maintained in darkness within sealed plastic boxes, at 22°C. All colonies contained male and female neotenic reproductives. Termites were considered true workers if they did not possess any sign of wing buds or distended abdomens. Termites were identified as *R. flavipes *by a combination of soldier morphology [69], and *16S *mitochondrial-ribosomal RNA gene sequencing [70]. The partial mitochondrial *16S *sequences of the four colonies used were deposited, respectively, in Genbank under accession numbers: FJ265704 (colony-1 "GB1"), FJ627943 (colony-2 "K2"), FJ265705 (colony-3 "A8") and GQ403073 (colony-4 "K5"). Using the *16S *mitochondrial sequences, colony 1 was 99% identical to mitochondrial haplotypes F22 and F1 (EU259755, EU259734), colony 2 was 98% identical to haplotype F20 (EU259753), colony 3 was 96% identical to haplotypes F34, 28, and 21 (EU259767, EU259761, EU259754) and colony 4 was 98% identical to haplotype F20 (EU259753).

### Phenotypic bioassays

Small-scale dish bioassays were conducted at 27°C as described previously [19,39]. Paired paper towel sandwiches were treated with acetone (controls), JH III, or SHE treatments delivered in acetone. JH III (75% purity; Sigma; St. Louis, MO) was provided at a rate of 112.5 μg per dish in a volume of 200 μl acetone. The JH III rate was chosen based on maximal efficacy with minimal mortality observed in previous concentration range studies [39]. After solvent evaporation, paper towel sandwiches were placed in 5 cm plastic Petri dishes and moistened with 150 μl of reverse osmosis water. Fifteen worker termites were placed in each assay dish. Live solider treatments consisted of holding two live soldiers with 15 workers from the same colony. Every five days, termites were counted, presoldier formation was noted, and water was added if needed. Each treatment was monitored for 25 days.

For larger-scale soldier inhibition assays, two treatments were examined to assess the influence of live solders on presoldier formation in large group format of 100 termites per dish. Control treatments included 100 workers only; whereas, soldier treatments included 90 workers + 10 soldiers. Termites from a single colony (K5) were used, and assays were run in large Petri dishes (9 cm diam.). Caste composition and survival were monitored every ten days for a total of 30 days. Each treatment was replicated five times and results pooled (avg ± SEM) for reporting.

### Preparation of solider head extracts

Soldier head extracts were prepared as described in Tarver et al. [19]. Soldiers (80-150 total) were isolated from lab colonies, and their heads removed and homogenized in 5 mL acetone using a Tenbroeck glass homogenizer. To remove particulate matter, the homogenate was fractionated by passing it through a glass Pasteur pipette filled with approximately 250 mg of silica gel (60-200 mesh) on top of a glass wool plug. The SHE was eluted with 10 column volumes of acetone and brought to 50 ml with acetone in a volumetric flask.

### Gene expression bioassays

A total of five different treatments were tested including acetone controls (300 μl), JH III (200 μl acetone containing 112.5 μg JH III), JH III+SHE (112.5 μg JH III in acetone + 1.5 soldier head equivalents in acetone), SHE (1.5 head equivalents extracted in acetone), and live soldiers (two per assay replicate). Each treatment was replicated five times for colony-1 and six times for colonies-2 and 3 (GB1, K2, and A8 respectively). Three biological replicates were used per treatment for colony 1 and four for colonies 2 and 3. Additional replicates for colonies 2 and 3 were added to improve statistical power. Samples of 15 termites were collected for destructive sampling at days 0, 1, 5, and 10. Collected samples were immediately frozen at -80°C.

### RNA isolation and cDNA synthesis

Total RNA was isolated from frozen samples using the SV total RNA Isolation System (Promega; Madison, WI) according to the manufacturer's protocol. Whole body RNA extracts were isolated from all 15 worker termites included in each bioassay dish. The amount of RNA was quantified by spectrophotometry and equal amounts of RNA were used in cDNA synthesis reactions. First-strand cDNA was synthesized using the iScript cDNA synthesis Kit (Bio-Rad; Hercules, CA) according to the manufacturer's protocol.

### Gene expression

The 49 candidate and reference genes were identified in recent *R. flavipes *sequencing projects and were chosen based on their homology to developmental or JH biosynthesis/metabolism genes [8,17,34,40,54,71]. The identity of all 49 PCR products was verified by direct sequencing. Quantitative real-time PCR (qRT-PCR) was performed using the iCycler iQ real-time PCR detection system (Bio-Rad) with SYBR-green product tagging (similar to [8,34]). cDNA, obtained as described above, was used as the qRT-PCR template. Gene specific primers are listed in Additional file 2: Table S2. Eleven total biological replicates were conducted for qRT-PCR (three from colony-1, and four each from colonies 2 and 3). Average Ct values of three technical replicates were pooled for analysis to represent each biological replicate.

### Reference gene selection

To select appropriate reference genes, all of the Ct values across all colonies, treatments, biological replicates, and technical replicates for each gene were analyzed to identify genes with the least amount of variation in expression (see [17,34]). Three genes with the lowest standard deviation were chosen for use as reference genes: *Stero-1*, *LIM*, and *Mev-1 *(Additional file 1: Table S1).

### Data and statistical analyses

Relative expression of target genes was calculated by comparing the average of the three technical replications first normalized to the reference genes and then normalized to the control treatment using the 2^-ΔCtΔCt ^method [72]. Normalized expression values (2^-ΔCtΔCt^) from all colony replicates were initially analyzed using the microarray visualization software ArrayStar (DNASTAR, Inc, Madison, Wisconsin, USA). To identify potential gene networks, genes with significant differential expression were clustered hierarchically using Euclidean distance metrics and centroid linkage for each day (1, 5 and 10) using ArrayStar.

To identify similarities between treatments, all genes were clustered hierarchically using Euclidean distance metrics and centroid linkage for each day (1, 5 and 10) using Array Star™ software.

To determine significantly differentially expressed genes, CT expression values for target genes were normalized to the CT values from the reference genes (ΔCT). A two-way ANOVA, with adjusted least squares (LS) means and false discovery rate (FDR) correction was used to distinguish significantly differentially expressed genes among treatments using JMP statistical software (SAS Institute, Cary, NC, USA) (Additional file 3: Table S3). Tukey's HSD test was used for separating means by treatment for each gene.

## Authors' contributions

MRT conceived the study design, performed all the experimental procedures and was the primary author of the manuscript. XZ participated in the design of the study. MES conceived the study design, analyzed data, and critically revised the manuscript. All the authors read and approved the final manuscript.

## Supplementary Material

Additional file 1 **Table S1**. Meta-analysis of all genes used to identify reference genes having the most stable expression. Genes highlighted in yellow are those with the most stable expression across time and treatments (i.e., smallest standard deviation; SD). Click here for file

Additional file 2 **Table S2**. Sequence accession numbers and quantitative real-time PCR primer details Click here for file

Additional file 3 **Table S3**. Summary of ANOVAs for each gene (down) and day (across). Click here for file

Additional file 4 **Table S4**. Day 1 relative expression values and summarized ANOVA results with FDR q-values Click here for file

Additional file 5 **Table S5**. Day 5 relative expression values and summarized ANOVA results with FDR q-values Click here for file

Additional file 6 **Table S6**. Day 10 relative expression values and summarized ANOVA results with FDR q-values Click here for file

Additional file 7 **Table S7**. Summary of horizontal gene clustering for Figures 2, 3 and 4. Click here for file
