# Supplementary material for: Socio-environmental and endocrine influences on developmental and caste-regulatory gene expression in the eusocial termite Reticulitermes flavipes
Source: BMC Mol Biol. 2010 Apr 23;11:28. doi: 10.1186/1471-2199-11-28 (PMC2873311; doi:10.1186/1471-2199-11-28)
Supplement: Additional file 1 — Table S1. Meta-analysis of all genes used to identify reference genes having the most stable expression. Genes highlighted in yellow are those with the most stable expression across time and treatments (i.e., smallest standard deviation; SD). [file 1471-2199-11-28-S1.DOC]

**Title: Table S1**

**Description: Meta-analysis of all genes used to identify reference genes having the most stable expression. Genes highlighted in yellow are those with the most stable expression across time and treatments (i.e., smallest standard deviation; SD).**
