# Supplementary material for: Socio-environmental and endocrine influences on developmental and caste-regulatory gene expression in the eusocial termite Reticulitermes flavipes
Source: BMC Mol Biol. 2010 Apr 23;11:28. doi: 10.1186/1471-2199-11-28 (PMC2873311; doi:10.1186/1471-2199-11-28)
Supplement: Additional file 2 — Table S2. Sequence accession numbers and quantitative real-time PCR primer details [file 1471-2199-11-28-S2.DOC]

**Title: Table S2**

**Description: Sequence accession numbers and quantitative real-time PCR primer details**
