# Supplementary material for: Socio-environmental and endocrine influences on developmental and caste-regulatory gene expression in the eusocial termite Reticulitermes flavipes
Source: BMC Mol Biol. 2010 Apr 23;11:28. doi: 10.1186/1471-2199-11-28 (PMC2873311; doi:10.1186/1471-2199-11-28)
Supplement: Additional file 3 — Table S3. Summary of ANOVAs for each gene (down) and day (across). [file 1471-2199-11-28-S3.DOC]

**Title: Table S3**

**Description: Summary of ANOVAs for each gene (down) and day (across).**
