# Supplementary material for: Socio-environmental and endocrine influences on developmental and caste-regulatory gene expression in the eusocial termite Reticulitermes flavipes
Source: BMC Mol Biol. 2010 Apr 23;11:28. doi: 10.1186/1471-2199-11-28 (PMC2873311; doi:10.1186/1471-2199-11-28)
Supplement: Additional file 5 — Table S5. Day 5 relative expression values and summarized ANOVA results with FDR q-values [file 1471-2199-11-28-S5.DOC]

**Title: Table S5**

**Description: Day 5 relative expression values and summarized ANOVA results with FDR q-values**
