# Supplementary material for: Socio-environmental and endocrine influences on developmental and caste-regulatory gene expression in the eusocial termite Reticulitermes flavipes
Source: BMC Mol Biol. 2010 Apr 23;11:28. doi: 10.1186/1471-2199-11-28 (PMC2873311; doi:10.1186/1471-2199-11-28)
Supplement: Additional file 7 — Table S7. Summary of horizontal gene clustering for Figures 2, 3 and 4. [file 1471-2199-11-28-S7.DOC]

**Title: Table S7**

**Description:** **Summary of horizontal gene clustering for Figures 2, 3 and 4.**
